# Medical students’ personal experience of high-stakes failure: case studies using interpretative phenomenological analysis

**DOI:** 10.1186/s12909-015-0371-9

**Published:** 2015-05-12

**Authors:** R.S. Patel, C. Tarrant, S. Bonas, R.L. Shaw

**Affiliations:** Department of Medical and Social Care Education, University of Leicester, Leicester, UK; Department of Health Sciences, University of Leicester, Leicester, UK; Department of Clinical Psychology, University of Leicester, Leicester, UK; School of Life & Health Sciences, Aston University, Birmingham, UK

**Keywords:** Underperformance, Failure, Experience, Medical student, Medical school, Interpretative phenomenological analysis

## Abstract

**Background:**

Failing a high-stakes assessment at medical school is a major event for those who go through the experience. Students who fail at medical school may be more likely to struggle in professional practice, therefore helping individuals overcome problems and respond appropriately is important. There is little understanding about what factors influence how individuals experience failure or make sense of the failing experience in remediation. The aim of this study was to investigate the complexity surrounding the failure experience from the student’s perspective using interpretative phenomenological analysis (IPA).

**Methods:**

The accounts of three medical students who had failed final re-sit exams, were subjected to in-depth analysis using IPA methodology. IPA was used to analyse each transcript case-by-case allowing the researcher to make sense of the participant’s subjective world. The analysis process allowed the complexity surrounding the failure to be highlighted, alongside a narrative describing how students made sense of the experience.

**Results:**

The circumstances surrounding students as they approached assessment and experienced failure at finals were a complex interaction between academic problems, personal problems (specifically finance and relationships), strained relationships with friends, family or faculty, and various mental health problems. Each student experienced multi-dimensional issues, each with their own individual combination of problems, but experienced remediation as a one-dimensional intervention with focus only on improving performance in written exams. What these students needed to be included was help with clinical skills, plus social and emotional support. Fear of termination of the their course was a barrier to open communication with staff.

**Conclusions:**

These students’ experience of failure was complex. The experience of remediation is influenced by the way in which students make sense of failing. Generic remediation programmes may fail to meet the needs of students for whom personal, social and mental health issues are a part of the picture.

## Background

A study at one UK University suggests that approximately 10-15 % of students fail to make satisfactory progress through medical school [1]. Failure at medical school has significant consequences for the students who fail, the faculty that provides support, and the institution that is responsible for delivering education. Students who struggle at medical school may be at risk of struggling as doctors [2, 3], therefore the problem also has implications for patients. Successful remediation for these struggling students is important.

The traditional method for academic remediation involves a 3-step process: identification of students who fail at assessment; a remediation period, usually repeating part of the course; and re-testing [4–6]. This approach is now under scrutiny since there is evidence that this merely perpetuates the problem and students continue to fail further along the course [6–9]. The 3-step approach is essentially ‘exam coaching’ [10] where the goal is to get the student over the hurdle of assessment, rather than to address the underlying problems that caused the student to fail.

Although students present with academic problems, these are often a proxy for other issues. These may be personal, professional or health-related, and consume substantial academic and pastoral care resources. Academic staff are beginning to recognize the importance of tailoring remediation approaches to the needs of the students with different types of problems. There have been efforts to develop profiles of struggling students such as the work of Hayes (2012), which identified problems arising from immaturity, learning skills, organizational skills, health or personal issues and poor insight. Such profiles can inform recommendations about appropriate interventions.

There is a risk, however, that pigeon-holing medical students into boxes atomizes the complexity of problems and may not lead to more accurate prognostication [10]. Furthermore, there is also concern that this type of profiling may be perceived as ‘medicalising’ the student experience of failure [10], whereas a more developmental approach in which attention is given to the individual over a greater length of time during the course rather than just around the failing episode would be more appropriate. There are few quantitative tools to help faculty fully understand the nature of problems among failing students, so a qualitative approach to the process of identifying the aetiology of failure may be helpful. The advantage of a model of diagnosis and management for a set of complex issues is that individuality can be preserved, such that no two individual ‘problem’ students are perceived as quite the same [10]. It follows that the management of remediation may be more effectively planned for the student.

Given the high admissions standards and competitive demand for medical school entry, successful students are typically high achievers. That can make encountering difficulty during medical training particularly challenging because it is at odds with their prior experience and developing sense of self. The aim of this study was to ‘bracket’ (i.e. put aside) (13) any predetermined notions of causes of failure and focus instead on first-hand accounts of medical students’ experiences of failure. The research questions were: “what is the experience of failing the final assessments at medical school from the student’s perspective?” and “what factors affect the way that students make sense of their remediation experience following failure?”

## Methods

### Approach

The value of qualitative methods in medical education and evidence-based medicine is clear [11, 12]; this paper demonstrates the added benefit of taking a case study approach by using the idiographic method, Interpretative Phenomenological Analysis (IPA) [13]. The conceptual framework of IPA is hermeneutic phenomenology [14]: hermeneutics is a theory of interpretation which puts meaning at the centre of the pursuit to understand human experience; phenomenology adopts an open-ended approach to describing phenomena in the world as they are experienced by individuals. A dialogic relationship between person and world is assumed which means that we cannot understand people in isolation and so must always take into account the context in which they live. In short, IPA research focuses on the person within their world in order to understand the phenomenon of interest from their perspective. IPA has been used to understand subjective responses to illness [15], identity formation [16], psychological distress [17], and well-being [18].

### The sample

The significance of case studies for making sense of the unique elements of experience is understood in clinical practice [19]. The same rationale is adopted here: we need both breadth and depth in science to enable ‘horizontal’ generalization from large (experimental) studies with representative samples (e.g. randomized controlled trials); but we also need to generalize conceptually (or ‘vertically’) from smaller samples of rich data for us to make sense of the variation between people living in different contexts [20]. Three ‘critical cases’ [21] were selected from a larger study carried out at two medical schools [22]. These demonstrate the incongruence of the experience of failure for these otherwise high achievers, and helped delineate the constituents of failure which students did not anticipate. These students had all failed their final-year examinations and requested to repeat the year in remediation. The students were invited to participate in the study at the start of their repeat year. Students were informed that involvement was voluntary and not a formal requirement by their medical school as a consequence of the failure. A formal consent procedure was undertaken after students had been given background information sheets about the study and assured of confidentiality.

### Data collection

Using semi-structured narrative interviews (see Appendix 1), participants were encouraged to describe their experiences of any failure on the course and how they made sense of these experiences. Although SB, CT and RP conducted the interviews, SB and CT were not involved with teaching of the students around the time of assessment or during remediation. Both SB and CT have a background in psychology and are lecturers in medical education. RP is a lecturer in medical education and provided informal teaching during the remediation period, however sessions were voluntary and not a formal part of the remediation programme for students at either medical school. Interviewers allowed participants to discuss topics and issues most relevant to them. Interviews lasted 60–90 min and were recorded on a digital recorder with the consent of participants and subsequently transcribed verbatim.

### Data analysis

The idiographic nature of IPA required transcripts to be analysed case-by-case within a 3-stage process that included an initial exploration of the data, a review of emergent themes and a final drawing together of themes across cases [23]. SB, CT, RP and RS analysed the data. RS has a background in psychology and is not a faculty member at either medical school in this study.

Initially, phenomenological coding involved a detailed examination of meaning units (small units of transcript). This was achieved by highlighting across sections of text and annotating the transcript with descriptive summaries of content, e.g. describing how participants made meaning from the failing experience. During this stage, notes were made of language, metaphor or imagery used by participants and the tone of their account [23].

The second stage involved interpretative coding: looking at the transcript as a whole to identify patterns or inconsistencies within the account. This included looking for repeated use of particular language, and asking questions about what this language told us about how the participant had made sense of the phenomena or how they presented themselves in relation to it. Emergent themes were noted on each of the three individual transcripts.

Finally, a cross-case analysis was conducted to generate a table of themes that represented the aspects of the phenomenon of failing which were shared between participants but also those aspects which were unique. Pseudonyms were used throughout data collection and analysis stages to preserve the anonymity of the participants.

### Reflexivity

The interpretative element of IPA involves an interaction between the researcher’s understanding of the phenomenon under investigation and the participant’s perceptions of the sense-making process [24]. Researchers are called upon to reflect on and make themselves aware of their own assumptions. This is to ensure their presuppositions (biases) do not impact unknowingly on the process of analysis and interpretation. The interviewers discussed findings among each other to challenge the trustworthiness of their own findings [25, 26]. RS provided an external perspective since she had no involvement with the participants or medical education.

### Ethics

The University of Leicester granted ethical approval for the study (EMCUF 6 26062013 SoM MEU), and reciprocal agreement was received from the University of Nottingham.

## Results

Two participants were female (Sarah and Alice) and one was male (Raj). Two of the three were graduate entrants (Sarah and Raj) and one was an undergraduate (Alice).

Analysis demonstrated how emotional and social factors are inextricably tied into students’ experiences of academic failure both in terms of causal influence on academic performance, and as sequelae to exam failures. We identified three themes: emotional trauma of struggling and failing; personal problems as inseparable from academic life; and social isolation. Other themes such as a lack of insight, poor self-regulated learning and external attributions for failure are described elsewhere [22]. We describe students’ experiences of failure below, of which remediation is a part.

### Emotional trauma of struggling and failing

Both Sarah and Alice struggled academically during their medical degree and failed a number of high stakes exams prior to failing finals. They both recognised limitations in their approaches to studying. Alice had dyslexia, and described how it took her a long time to read and assimilate information; she also felt that she tried to go into too much depth in her revision. Sarah also recognised that her revision was not sufficiently focused. The most notable feature of their accounts of struggling and failing, however, was their description of the anxiety and emotional trauma they experienced.

Sarah had successfully completed a previous degree in a different subject, and was shocked to find that she struggled at medical school. She experienced anxiety about failure in exams, describing a fear of ‘looking stupid’. Clinical exams in particular challenged her ability to protect her self-esteem in front of others, since her low ability was there for all to see.

*In the [clinical exam] there's the opportunity to look like a real idiot […] ‘cos in my mind I'm really trying to focus but I'm always going 'Don't look like an idiot, don't look like an idiot’ which obviously is counterproductive but I seem unable to switch it off [Sarah]*

Sarah’s account suggests that she found it very hard to come to terms with this new identity of being a ‘struggling’ student. She believed she was always one mark off or one question away from passing the written papers during the course, and this perception of ‘just failing’ drove her to keep going in order to ‘get over the line’.

*It was like really close, I remember that was partly why it was so devastating ‘cos I was like 1 station away and I think it was 1 question as well on the written […] So I was kicking myself [Sarah]*

She felt that success just required her to work harder, but the pressure eventually took its toll and she developed depression. She failed the high-stakes mid-course exam and had to repeat the year. She described feeling ‘numb’ in the wake of failure. Her ability to think rationally was affected, she lost motivation for learning and felt she had sabotaged her potential because of her low mood and lack of motivation.

*Truthfully I lost motivation […] ‘cos I'd worked so hard [previously …] and I still didn't make it through […] so I was like ‘Argh, what was the difference? I'm either gonna fail by 1 mark or catastrophically, it doesn't make any difference’ […] In finals the first time round I hadn’t done enough work. I'd self-sabotaged with my 'What's the point?’ [Sarah]*

Alice also had high expectations of herself, having been a successful student prior to medical school. She described very similar anxieties to Sarah about the risk to her self-esteem from performing poorly in exams, particularly clinical exams. The drive to maintain her well-being prevented her from developing the resilience required for performing at assessment and learning how to think clearly under pressure.

*I will never forget that, it was a diabetes station and the demonstrator asked me a question and I wasn’t sure. She just told me to ‘think’. […] She sat there just looking at me and I was just trying to think. I said ‘I don’t know, can I move on? And then she just looked at me, gave me this stupidest, weirdest look. It was really horrible [Alice]*

Alice was devastated by her exam failures, which severely undermined her sense of self-worth. In describing her experiences, she used language suggestive of trauma and post-traumatic stress.

*I dreamt about [the exam]. I had nightmares. […] That day, what I was wearing, you know I didn’t wear any of those clothes again. I stopped using the perfume […] it was awful [Alice]*

She described feelings of guilt at letting her family down, and became increasingly fearful of failure. Her experience of repeated failures, and associated fear and trauma resulted in a downward slide into severe depression, making it difficult for her to engage with the course, or to face sitting an exam.

*I would rather have died than do that exam where I would go and for a whole, I couldn’t bear you know sitting there for I don’t know 8 hour, 10 hours and then the same the following day. I couldn’t [Alice]*

Raj’s first experience of failure came at finals. He described how it had taken him some time to come to terms with the emotional fall-out of failing, but, because he could attribute this failure to breakdowns in his personal life rather than his academic ability, he was better able to make sense of the failure and move forward.

*I was gutted. Completely gutted […] It was a surprise in some sense and not in others. I still don’t think my head was fully with it to be honest. […] It was a blur then but I know I feel totally differently now to what I did then [Raj]*

### Personal problems as inseparable from academic life

Personal problems affected all of the participants and all of them failed to cope with them whilst trying to prepare for assessment.

Sarah struggled academically throughout the course and experienced ill health and depression. These problems were compounded by relationship breakdowns at the time of a mid-course high-stakes exam, and again around finals, which impacted negatively on her ability to study and her mental health.

*[Then] another relationship breakdown actually […] The weekend before finals […] I remember the day before finals I was on the floor crying over it […] I just couldn't focus [Sarah]*

She also experienced financial hardship and worried more about making ends meet. One consequence of this was she did not take her depression medication regularly.

*I was on treatment [for depression] on and off. Ridiculously because I didn't have the money, […] I actually didn't have £7 for a prescription so I sort of came off without supervision. It was entirely the cost [Sarah]*

Sarah felt that she had to keep these problems to herself rather than sharing them with, or seeking help via, the Medical School. She made a stark contrast between what she believed was the position of the medical school – that students should keep their personal problems separate to their academic life – and her own belief that the emotional consequences of personal problems inevitably impacted on her ability to succeed. This meant that in her appeal following failure she played down what she felt was the ‘real’ reason for failure, and instead worked to create an account that she felt would be more acceptable to the official panel. Interestingly her account demonstrates a belief that health problems, but not personal problems, would be judged to be legitimate reasons for failure.

*I know medical school isn't interested in relationships so in my appeal had to emphasise some of the other problems I had going on, but really it was the relationship that caused me such problems. I think if it had been just a few health problems, I don't think I would have descended into depression again.*

For Alice, personal problems were not the most prominent feature of her account, but her experiences of struggling academically were set in context of a bereavement.

*It was a good start to Phase 2, but then unfortunately my best friend from back home died in a car accident […]. I think I cried for 2 months, I cried every time I saw a patient […] I couldn’t study […] I missed 2 months [of the course] [Alice]*

While Alice suffered serious mental health problems as a result of her experiences of failure, as described in the previous section, her account suggested that the loss of her friend acted as a tipping point, resulting in the development of severe depression.

*I was going downhill, and just pushed myself, it didn’t affect my studies really until around December […] I was so depressed I couldn’t get out of bed […] I had severe depression, at times I thought of ending my life, I missed my friend so much [Alice]*

Alice approached the Medical School about her depression, and accessed counselling. She found this helpful in coping with her feelings, but found counselling sessions exhausting, leaving her unable to study while she recovered from each session.

*I wanted the counselling and it really helped, but it took a lot out of me. […] It would knock me out for a whole 2 days […] I’d be in bed. I was so tired emotionally and drained [Alice]*

Personal problems were the main reasons implicated in Raj’s experience of failure. Having been a successful student throughout his medical degree, he experienced a relationship breakdown before his final exams, resulting in a downward spiral of emotional and financial problems. He found this period of time extremely traumatic and distressing, and described how things fell apart for him.

*[My partner] left about 3, 4 months before Finals. We’d got a house together and she just disappeared and left me with all the bills. I was about three thousand pounds in debt [and I] suddenly owed the landlord [that money]. [He] basically evicted me. […] I hadn’t got any money, my insurance didn’t go out for my car. I didn’t realise it hadn’t gone out so they caught me and then they banned me driving. […] My head was all over the place [Raj]*

Raj felt that his motivation and ability to study were damaged.

*My mood must have been very low and you know and I just wasn’t focussed on it and even when it came to revising for Finals. I didn’t really make any active steps I just sort of sat there with a book in front of me and there was no real sort of drive to go and strive [Raj]*

Raj’s view of the Medical School’s attitude to personal problems echoed those of Sarah. Although he accessed support through Central University services, he didn’t feel he could discuss personal problems with his personal tutor, or other staff in the Medical School.

*They're not really that interested to be perfectly honest, so not from my experience, they didn’t seem that interested. […] So I didn’t really have a brilliant experience with personal tutors. […] I didn’t go anywhere else, only sort of like close network of friends [Sarah]*

### Social isolation

All three students recognised in hindsight that the ability to draw on the support of other students and engage in group study was critical for success. The students felt that they had been relatively socially isolated for a variety of reasons, and that this had disadvantaged them.

Sarah believed making friends was difficult for her since she was older than her peers. She already had a group of ‘best friends’ from her previous University experience and did not invest effort in trying to make new friends on her medical course

*I was 20-something when I started and everyone else was 18 and I suppose even thought I probably wasn’t that interested in some of their things, I had a different attitude, I wouldn’t get invited to the same things [Sarah]*

Sarah acknowledged that she had missed opportunities to benefit from social learning, and she reflected on this in considering what advice she would give herself if she was starting her course now.

*You can’t pass medicine alone. There’s too much. […] Remember that you can’t study medicine alone so make time for the socialising because you need to get into those revision groups [Sarah]*

Alice had support from her boyfriend who was also studying medicine, but struggled with finding enough time to make friends, in the context of the amount of time and effort she felt she had to put into study.

*I never studied with anyone. […] I went home in December then my exams of the first term were in January and throughout all this time besides going home to my family I really did not go out anywhere [Alice]*

She felt that the culture at Medical School was such that it was difficult to discuss failing with other students, meaning that she ended up working for resits alone.

*Medical students […] don’t wanna tell others that they’ve failed, so even though I knew a couple who had failed like me, everyone wanted to be a 100 % private […] I’m not gonna approach any of the other students, they’d probably think I was weird or something so I didn’t, so I was alone [Alice]*

Raj felt that he had had a good social network during his studies, but that this network had dispersed by the time he discovered he had failed his finals, leaving him to face this alone.

*I didn’t really have [any support]. A lot of my friends that I had in [city], they’d all dropped out, or failed or gone to different places and things, so I wasn’t left with massive amounts of friends like towards the end [Raj]*

### Remediation

Sarah failed both the written and clinical elements of the final exams. At the time of interview Sarah had not started remediation, but her relationship with the Medical School had been damaged by her perceptions of the response to her failure; she did not believe that the Medical School was motivated to act in her best interests.

*I didn’t make it through the first appeal [to return after failing finals], got through on the second appeal. […] I don’t know if this is true but this is another rumour circulating round, that we've got too many students […] so it’s in their interest to kick you out [Sarah]*

At finals, Alice passed the written papers, but failed the clinical exams. By the time of resits she was suffering from insomnia, anxiety and panic attacks. Alice felt that the generic support provided during remediation, which focused on approaches to learning, was not appropriate for her.

*It was about how we study, how we can improve, there was some exercises. To me I found them really patronising because […] I hadn’t failed the written [exam] and they focused a lot about the written and they wanted to identify any study, any learning problems […] The thing is for me I don’t have any of them. […] My needs were ‘are you okay, how are you feeling?’ [Alice]*

She appreciated the attention and support that she received from her personal tutor during the remediation period, but her account had an underlying tone of bitterness and regret that the Medical School had not given her the same level of support and care during her degree, which may have helped her to get through without failing.

*They did pay a lot of attention to us this year […], but I think it’s too late now. Now that I’ve failed, you’ve wanted to care about how I feel. How about before that? It could have not ever happened if you cared or gave a bit more of attention [Alice]*

Raj, having failed due to personal rather than academic difficulties, found the generic nature of remediation frustrating.

*It’s quite generic. […] We’ve got very specific problems […] and I just found that nobody sort of said ‘here’s your problems, how do you think we can deal with these problems?’ [Raj]*

He described how his own attempts to proactively seek support that met his needs during the remediation period had been met with a punitive response from the Medical School. He felt that this was unfair, and reflected assumptions by the Medical School that failing students were inherently ‘bad’ and untrustworthy. This experience acted as a barrier to open dialogue about how remediation could be tailored to his needs.

*I missed 1 teaching session and I went to another one because I thought I’d get more out of the other one. […] Instead of turning around and saying ‘You felt like you had to go somewhere else, why? Is there anything we could do to make that better?’ [the Medical School…] slapped the best efforts letter on you. […] To be honest I’d got a bit scared to ask after that [Raj]*

## Discussion

This work has demonstrated the individualised journeys of failing students, within which there may be common themes. We have been able to demonstrate how the interaction between internal and external factors may overwhelm students who fail, thus hindering or preventing their remediation. Students attributed their failure, and subsequent emotional experiences, to a range of internal and external factors, including their own abilities, and health and personal problems. External attributions for failure were found to be a coping mechanism in another larger study of medical students’ experiences of failure [22].

The analysis identified that failing in itself can generate significant emotional trauma. For these students, personal problems and academic difficulties were intimately interlinked, but students felt an expectation that these should be hidden from the world of medical training because they were categorized as ‘disavowed’ within idealized constructs of the medical profession [27]. This made it difficult for them to access support and suggests that students need to develop emotional intelligence and self-awareness to recognise the links between personal growth (and struggles) and their professional competence. Boosting students’ self-efficacy (confidence to achieve specific tasks) [28] for reflective learning alongside developing their professional confidence would provide the means through which personal struggles can be legitimised and dealt with appropriately [29].

These students also experienced social isolation, which deprived them of opportunities for social learning. Students’ experiences of failing had significant consequences for their relationship with the Medical School and reactions to the remediation support provided. The study findings have implications for the ways in which medical schools seek to understand, support and remediate students.

This study provides further evidence that personal problems [30], poor relationships with others and mental health problems [31, 32] can arise from, and can complicate, academic difficulties. Despite this complexity, the majority of remediation still involves the provision of more teaching [33]. This study suggests providing yet more teaching to students with complex problems will not be effective for managing unresolved personal problems, developing interpersonal skills or improving psychological well-being. Likewise, a remediation programme that delivers ‘more of the same’ probably comes about from the perception that the ‘same’ students fail for the ‘same’ reasons [34, 35] each year. The findings challenge such a perception and raise the concern surrounding the usefulness of ‘one-size fits all’ remediation programmes for managing complex failure such as the type encountered at re-sit finals.

There are systems in place for classifying and managing the students who fail at assessment based on whether individuals struggle for predominantly academic, personal or professional [1, 36] reasons. The findings from this study suggest this classification remains simplistic since the ‘reality’ for many students is a more complex interaction between several factors, all of which may conspire to cause failure. Yates [36] recognised the value of recording adverse health, social and behavioural events encountered by students to better predict those at risk of failure and this study adds more support for that to happen. Whilst those studies used quantitative methods for better understanding the problem of failure prior to it happening, this study attempted to explore the nature of the problem after the event through the use of a qualitative approach.

Stress and vulnerability are recognised among medical students at the start the course [37], however the findings from this study suggest both may be present in significantly high levels among students susceptible to repeated failure. The issues of vulnerability raises particular concerns about the systems for evaluating and managing student needs in remediation since the role of remediator may be compromised if the individual is perceived as both ‘judge and jury’ by the student. The preparedness of students to disclose these issues in this study proposes the need to ‘firewall’ the responsibility of remediation to individuals with a sole responsibility for remediation only, rather than one that is orientated towards performance management and preparing a case for removing the student from the course.

### Strengths and limitations of the study

The use of an IPA approach is a particular strength of this study; this approach has generated an important perspective on the experience of failing students, and one that differs in depth and focus from that generated by using a thematic analysis approach. The idiographic nature of IPA enables the individual story to be preserved, giving vivid and cohesive pictures of the experiences of individual students. There is potential for the essence of the IPA technique to be transported to an educational setting. A simple recommendation might be that education providers offer failing students a safe, one-to-one meeting with a personal tutor or equivalent to reflect upon their performance and their situation. The process of articulating this will evoke meaning-making, which if facilitated in a non-judgmental way will identify idiosyncratic issues and enable the development of individualised strategies for remediation.

This study presents findings from a small sample of only three students. This is consistent with recommendations for studies using IPA to gain a deeper understanding of a phenomenon [13]. This approach, while enabling a deep and rich understanding of the accounts of individual students, has some limitations. The students in this study had failed finals and the re-sits, and the experience of failure may be different for students who fail other high-stakes assessments but subsequently pass and graduate as doctors. Alongside the experiences of failing students, the perceptions of faculty and other students who don’t experience failure would help provide a deeper understanding of the phenomenon. The experience of failing is presented from a UK context in two Universities, and the phenomenon may be different within other institutions or from an international perspective.

## Conclusions

The use of IPA uncovered the complexity of these students’ experiences of failure, and highlighted the extent to which the experience of remediation is influenced by the way in which students make sense of failing. The findings explain why generic remediation programmes may fail to meet the needs of students for whom personal, social and mental health issues are part of the picture.

## Appendix 1

### The semi-structured student interview topic guide

Demographics … where did they study before coming to medical school?

On arrival at Medical school …

What were your expectations of getting through assessments

What did you do to prepare yourself?

How did you feel about exams – preparing, going in, afterwards?

The first time you were un-satisfactory in an exam …

What happened? What did you do?

How did you feel?

How did you experience the Medical School’s response?

What support was available? What did you take up/not take up? Why?

What helped? What didn’t help?

What happened next with regard to assessment?

What happened? What did you do?

How did you feel?

How did you experience the Medical School’s response?

What support was available? What did you take up/not take up? Why?

What helped? What didn’t help?

With hindsight, what do you think lead up to you struggling with assessment?

What would you change if you could about …

How you did things

How the Med School did things

Anything else

If you think of anything else you want to say, you can raise things in the focus group – but there maybe things that you prefer not to talk about in a group – so is there anything that you would like to add to what you have said today?

## Abbreviations

IPA: Interpretative Phenomenological Analysis
UK: United Kingdom
